# Supplementary material for: Characterization of a CXCR4 antagonist TIQ-15 with dual tropic HIV entry inhibition properties
Source: PLoS Pathog. 2024 Aug 15;20(8):e1012448. doi: 10.1371/journal.ppat.1012448 (PMC11349218; doi:10.1371/journal.ppat.1012448)
Supplement: S1 Data — (DOCX) [file ppat.1012448.s008.docx]

**Source data for Fig. 1C**

|  | **+ SDF-1a** | **- SDF-1a** | +TIQ-15  **50 uM** | +TIQ-15  **10 uM** | +TIQ-15  **2 uM** | +TIQ-15  **400 nM** | +TIQ-15  **80 nM** | +TIQ-15  **16 nM** | +TIQ-15  **3.2 nM** | +TIQ-15  **640 pM** |
| --- | --- | --- | --- | --- | --- | --- | --- | --- | --- | --- |
| **Migrating Cells** | 72930 | 2500 | 0  (blow 2500) | 3060 | 13260 | 23460 | 47430 | 65280 | 66300 | 71400 |

**Source data for Fig. 3B**

|  | **Cell only** | **+HIV** | +TIQ-15  **10 uM** | +TIQ-15  **2 uM** | +TIQ-15  **400 nM** | +TIQ-15  **80 nM** | +TIQ-15  **16 nM** | +TIQ-15  **3.2 nM** | +TIQ-15  **640 pM** | +TIQ-15  **128 pM** | +TIQ-15  **25.6 pM** |
| --- | --- | --- | --- | --- | --- | --- | --- | --- | --- | --- | --- |
| **Luc reading**  **(x 1,000)** | 62.2 | 20,830 | 927.1 | 3,667 | 3,642 | 5,053 | 12,030 | 13,070 | 25,570 | 25,300 | 26,650 |

**Source Data for Fig. 3D**

Donor 1, HIV p24 (ng/ml)

|  | **Control** | + TIQ-15  **50 uM** | + TIQ-15  **10 uM** | + TIQ-15  **2 uM** | + TIQ-15  **400 nM** | + TIQ-15  **80 nM** | + TIQ-15  **16 nM** |
| --- | --- | --- | --- | --- | --- | --- | --- |
| Day 6 | 0.34 | 0.33 | 0.2 | 0.24 | 0.28 | 0.18 | 0.18 |
| Day 8 | 14 | 0.29 | 0.41 | 0.62 | 0.71 | 2.38 | 15.69 |
| Day 10 | 156.75 | 0.3 | 0.56 | 16.57 | 52.87 | 69.03 | 111.73 |

Donor 2, HIV p24 (ng/ml)

|  | **Control** | + TIQ-15  **10 uM** | + TIQ-15  **2 uM** | + TIQ-15  **400 nM** | + TIQ-15  **80 nM** | + TIQ-15  **16 nM** |
| --- | --- | --- | --- | --- | --- | --- |
| Day 6 | 1.31 | 0.27 | 0.26 | 0.26 | 0.26 | 0.27 |
| Day 8 | 7.28 | 0.25 | 0.36 | 0.71 | 0.96 | 5.02 |
| Day 10 | 105.4 | 0.25 | 1.27 | 13.29 | 29.72 | 74.01 |
| Day 12 | 150.34 | 0.26 | 4.27 | 64.22 | 85.9 | 125.24 |
| Day 14 | 255.79 | 0.3 | 11.71 | 134.43 | 173.87 | 192.96 |

Donor 3, HIV p24 (ng/ml)

|  | **Control** | + TIQ-15  **10 uM** | + TIQ-15  **2 uM** | + TIQ-15  **400 nM** | + TIQ-15  **80 nM** | + TIQ-15  **16 nM** |
| --- | --- | --- | --- | --- | --- | --- |
| Day 5 | 0.48 | 0.34 | 0.32 | 0.34 | 0.31 | 0.39 |
| Day 6 | 0.58 | 0.36 | 0.39 | 0.37 | 0.28 | 0.41 |
| Day 8 | 20.85 | 0.34 | 0.51 | 0.8 | 3.15 | 15 |
| Day 10 | 83.69 | 0.28 | 5.01 | 31.75 | 45.35 | 65.23 |

**Source data for Table 1.**

TIQ-15 in a Panel of Clinical Isolates

INHIBITION OF HIV-1 00KE_KER2008 REPLICATION IN PBMC BY TIQ-15

| **RT Values(cpm)** | | | | | | | | |  |  |  |
| --- | --- | --- | --- | --- | --- | --- | --- | --- | --- | --- | --- |
| **CONC (μM)** | **0.0** | **0.0003** | **0.001** | **0.006** | **0.03** | **0.16** | **0.80** | **4.00** | | **20** | **100** |
| **SAMPLE1** | 19836 | 23866 | 26717 | 20310 | 28061 | 19982 | 9761 | 2352 | | 229 | 69 |
| **SAMPLE2** | 20653 | 20910 | 30239 | 18395 | 16185 | 19313 | 11234 | 1191 | | 81 | 61 |
| **SAMPLE3** | 24763 | 22986 | 23504 | 15817 | 14533 | 19444 | 8086 | 1263 | | 33 | 65 |
| **M EA N** | 21750.3 | 22587.3 | 26820.0 | 18174.0 | 19593.0 | 19579.7 | 9693.7 | 1602.0 | | 114.3 | 65.0 |

| **TOXICITY VALUES (Cell Titer 96 - O. D. @ 490/650 nm)** | | | | | | | | | | |
| --- | --- | --- | --- | --- | --- | --- | --- | --- | --- | --- |
| **CONC (μM)** | **0.0** | **0.0003** | **0.001** | **0.006** | **0.03** | **0.16** | **0.80** | **4.00** | **20** | **100** |
| **SAMPLE1** | 0.728 | 0.764 | 0.714 | 0.777 | 0.753 | 0.924 | 0.981 | 0.996 | 0.611 | 0.076 |
| **SAMPLE2** | 0.906 | 0.877 | 0.901 | 0.919 | 0.838 | 0.966 | 1.065 | 1.043 | 0.632 | 0.077 |
| **SAMPLE3** | 0.944 | 0.952 | 0.960 | 0.959 | 0.944 | 1.055 | 1.243 | 1.110 | 0.656 | 0.077 |
| **M EA N** | 0.859 | 0.865 | 0.858 | 0.885 | 0.845 | 0.981 | 1.096 | 1.050 | 0.633 | 0.077 |

**INHIBITION OF HIV-1 91US001 REPLICATION IN PBMC BY TIQ-15**

| **RT Values(cpm)** | | | | | | | | |  |  |  |
| --- | --- | --- | --- | --- | --- | --- | --- | --- | --- | --- | --- |
| **CONC (μM)** | **0.0** | **0.0003** | **0.001** | **0.006** | **0.03** | **0.16** | **0.80** | **4.00** | | **20** | **100** |
| **SAMPLE1** | 19326 | 19231 | 31129 | 19151 | 21914 | 17217 | 12534 | 2407 | | 100 | 124 |
| **SAMPLE2** | 19692 | 21792 | 28444 | 21489 | 13914 | 17513 | 8264 | 1210 | | 72 | 108 |
| **SAMPLE3** | 17428 | 19538 | 27127 | 25950 | 14780 | 16708 | 7137 | 1454 | | 0 | 4 |
| **M EA N** | 18815.3 | 20187.3 | 28900.3 | 22197.0 | 16869.7 | 17146.3 | 9312.0 | 1690.7 | | 57.6 | 79.0 |

| **TOXICITY VALUES (Cell Titer 96 - O. D. @ 490/650 nm)** | | | | | | | | | | |
| --- | --- | --- | --- | --- | --- | --- | --- | --- | --- | --- |
| **CONC (μM)** | **0.0** | **0.0003** | **0.001** | **0.006** | **0.03** | **0.16** | **0.80** | **4.00** | **20** | **100** |
| **SAMPLE1** | 0.728 | 0.764 | 0.714 | 0.777 | 0.753 | 0.924 | 0.981 | 0.996 | 0.611 | 0.076 |
| **SAMPLE2** | 0.906 | 0.877 | 0.901 | 0.919 | 0.838 | 0.966 | 1.065 | 1.043 | 0.632 | 0.077 |
| **SAMPLE3** | 0.944 | 0.952 | 0.960 | 0.959 | 0.944 | 1.055 | 1.243 | 1.110 | 0.656 | 0.077 |
| **M EA N** | 0.859 | 0.865 | 0.858 | 0.885 | 0.845 | 0.981 | 1.096 | 1.050 | 0.633 | 0.077 |

**INHIBITION OF HIV-1 98US_MSC5016 REPLICATION IN PBMC BY TIQ-15**

| **RT Values(cpm)** | | | | | | | | |  |  |  |
| --- | --- | --- | --- | --- | --- | --- | --- | --- | --- | --- | --- |
| **CONC (μM)** | **0.0** | **0.0003** | **0.001** | **0.006** | **0.03** | **0.16** | **0.80** | **4.00** | | **20** | **100** |
| **SAMPLE1** | 23058 | 29276 | 32008 | 20623 | 29609 | 16504 | 9012 | 1934 | | 372 | 39 |
| **SAMPLE2** | 26912 | 22694 | 26583 | 25510 | 20797 | 18094 | 11257 | 1353 | | 164 | 0 |
| **SAMPLE3** | 25386 | 24360 | 25383 | 30521 | 21792 | 23315 | 12639 | 2031 | | 0 | 0 |
| **M EA N** | 25118.5 | 25442.8 | 27990.8 | 25550.8 | 24065.5 | 19303.8 | 10968.8 | 1772.2 | | 178.3 | 12.8 |

| **TOXICITY VALUES (Cell Titer 96 - O. D. @ 490/650 nm)** | | | | | | | | | | |
| --- | --- | --- | --- | --- | --- | --- | --- | --- | --- | --- |
| **CONC (μM)** | **0.0** | **0.0003** | **0.001** | **0.006** | **0.03** | **0.16** | **0.80** | **4.00** | **20** | **100** |
| **SAMPLE1** | 0.728 | 0.764 | 0.714 | 0.777 | 0.753 | 0.924 | 0.981 | 0.996 | 0.611 | 0.076 |
| **SAMPLE2** | 0.906 | 0.877 | 0.901 | 0.919 | 0.838 | 0.966 | 1.065 | 1.043 | 0.632 | 0.077 |
| **SAMPLE3** | 0.944 | 0.952 | 0.960 | 0.959 | 0.944 | 1.055 | 1.243 | 1.110 | 0.656 | 0.077 |
| **M EA N** | 0.859 | 0.865 | 0.858 | 0.885 | 0.845 | 0.981 | 1.096 | 1.050 | 0.633 | 0.077 |

**INHIBITION OF HIV-1 92UG046 REPLICATION IN PBMC BY TIQ-15**

| **RT Values(cpm)** | | | | | | | | | | |  |
| --- | --- | --- | --- | --- | --- | --- | --- | --- | --- | --- | --- |
| **CONC (μM)** | **0.0** | **0.0003** | **0.001** | **0.006** | **0.03** | **0.16** | **0.80** | **4.00** | **20** | **100** | |
| **SAMPLE1** | 16362 | 19200 | 6015 | 715 | 366 | 244 | 1831 | 307 | 66 | 186 | |
| **SAMPLE2** | 22085 | 15364 | 3074 | 395 | 16 | 16 | 130 | 86 | 42 | 50 | |
| **SAMPLE3** | 17905 | 7780 | 15304 | 1069 | 589 | 189 | 227 | 118 | 42 | 0 | |
| **M EA N** | 18783.8 | 14114.2 | 8130.5 | 725.8 | 323.2 | 149.2 | 728.8 | 169.8 | 49.5 | 78.3 | |

| **TOXICITY VALUES (Cell Titer 96 - O. D. @ 490/650 nm)** | | | | | | | | | | |
| --- | --- | --- | --- | --- | --- | --- | --- | --- | --- | --- |
| **CONC (μM)** | **0.0** | **0.0003** | **0.001** | **0.006** | **0.03** | **0.16** | **0.80** | **4.00** | **20** | **100** |
| **SAMPLE1** | 0.728 | 0.764 | 0.714 | 0.777 | 0.753 | 0.924 | 0.981 | 0.996 | 0.611 | 0.076 |
| **SAMPLE2** | 0.906 | 0.877 | 0.901 | 0.919 | 0.838 | 0.966 | 1.065 | 1.043 | 0.632 | 0.077 |
| **SAMPLE3** | 0.944 | 0.952 | 0.960 | 0.959 | 0.944 | 1.055 | 1.243 | 1.110 | 0.656 | 0.077 |
| **M EA N** | 0.859 | 0.865 | 0.858 | 0.885 | 0.845 | 0.981 | 1.096 | 1.050 | 0.633 | 0.077 |

**INHIBITION OF HIV-1 CMU02 REPLICATION IN PBMC BY TIQ-15**

| **RT Values(cpm)** | | | | | | | | | | |
| --- | --- | --- | --- | --- | --- | --- | --- | --- | --- | --- |
| **CONC (μM)** | **0.0** | **0.0003** | **0.001** | **0.006** | **0.03** | **0.16** | **0.80** | **4.00** | **20** | **100** |
| **SAMPLE1** | 13164 | 12849 | 5828 | 580 | 239 | 260 | 33 | 215 | 203 | 0 |
| **SAMPLE2** | 13988 | 10034 | 17476 | 12548 | 289 | 180 | 125 | 135 | 39 | 7 |
| **SAMPLE3** | 14076 | 10765 | 3137 | 4670 | 96 | 251 | 0 | 0 | 3 | 0 |
| **M EA N** | 13742.5 | 11215.7 | 8813.3 | 5932.3 | 207.7 | 230.0 | 52.4 | 116.4 | 81.3 | 2.2 |

| **TOXICITY VALUES (Cell Titer 96 - O. D. @ 490/650 nm)** | | | | | | | | | | |
| --- | --- | --- | --- | --- | --- | --- | --- | --- | --- | --- |
| **CONC (μM)** | **0.0** | **0.0003** | **0.001** | **0.006** | **0.03** | **0.16** | **0.80** | **4.00** | **20** | **100** |
| **SAMPLE1** | 0.728 | 0.764 | 0.714 | 0.777 | 0.753 | 0.924 | 0.981 | 0.996 | 0.611 | 0.076 |
| **SAMPLE2** | 0.906 | 0.877 | 0.901 | 0.919 | 0.838 | 0.966 | 1.065 | 1.043 | 0.632 | 0.077 |
| **SAMPLE3** | 0.944 | 0.952 | 0.960 | 0.959 | 0.944 | 1.055 | 1.243 | 1.110 | 0.656 | 0.077 |
| **M EA N** | 0.859 | 0.865 | 0.858 | 0.885 | 0.845 | 0.981 | 1.096 | 1.050 | 0.633 | 0.077 |

**INHIBITION OF HIV-1 93BR020 REPLICATION IN PBMC BY TIQ-15**

| **RT Values(cpm)** | | | | | | | | | | |
| --- | --- | --- | --- | --- | --- | --- | --- | --- | --- | --- |
| **CONC (μM)** | **0.0** | **0.0003** | **0.001** | **0.006** | **0.03** | **0.16** | **0.80** | **4.00** | **20** | **100** |
| **SAMPLE1** | 16163 | 310 | 169 | 133 | 318 | 142 | 45 | 164 | 116 | 52 |
| **SAMPLE2** | 22248 | 36 | 78 | 41 | 36 | 78 | 24 | 44 | 8 | 28 |
| **SAMPLE3** | 17567 | 90 | 69 | 24 | 167 | 57 | 62 | 64 | 68 | 20 |
| **M EA N** | 18659.2 | 145.3 | 105.3 | 66.0 | 173.7 | 92.3 | 43.7 | 90.7 | 64.0 | 33.3 |

| **TOXICITY VALUES (Cell Titer 96 - O. D. @ 490/650 nm)** | | | | | | | | | | |
| --- | --- | --- | --- | --- | --- | --- | --- | --- | --- | --- |
| **CONC (μM)** | **0.0** | **0.0003** | **0.001** | **0.006** | **0.03** | **0.16** | **0.80** | **4.00** | **20** | **100** |
| **SAMPLE1** | 0.728 | 0.764 | 0.714 | 0.777 | 0.753 | 0.924 | 0.981 | 0.996 | 0.611 | 0.076 |
| **SAMPLE2** | 0.906 | 0.877 | 0.901 | 0.919 | 0.838 | 0.966 | 1.065 | 1.043 | 0.632 | 0.077 |
| **SAMPLE3** | 0.944 | 0.952 | 0.960 | 0.959 | 0.944 | 1.055 | 1.243 | 1.110 | 0.656 | 0.077 |
| **M EA N** | 0.859 | 0.865 | 0.858 | 0.885 | 0.845 | 0.981 | 1.096 | 1.050 | 0.633 | 0.077 |

**INHIBITION OF HIV-1 JV1083 REPLICATION IN PBMC BY TIQ-15**

| **RT Values(cpm)** | | | | | | | | | | |
| --- | --- | --- | --- | --- | --- | --- | --- | --- | --- | --- |
| **CONC (μM)** | **0.0** | **0.0003** | **0.001** | **0.006** | **0.03** | **0.16** | **0.80** | **4.00** | **20** | **100** |
| **SAMPLE1** | 13974 | 22593 | 24036 | 23025 | 16756 | 15963 | 10006 | 3072 | 420 | 115 |
| **SAMPLE2** | 13420 | 20609 | 14540 | 19924 | 17758 | 17337 | 10365 | 2423 | 199 | 163 |
| **SAMPLE3** | 16502 | 14033 | 18777 | 11847 | 18168 | 16547 | 8254 | 1649 | 155 | 51 |
| **M EA N** | 14631.7 | 19077.8 | 19117.2 | 18264.8 | 17560.2 | 16615.2 | 9541.2 | 2380.8 | 257.5 | 109.2 |

| **TOXICITY VALUES (Cell Titer 96 - O. D. @ 490/650 nm)** | | | | | | | | | | |
| --- | --- | --- | --- | --- | --- | --- | --- | --- | --- | --- |
| **CONC (μM)** | **0.0** | **0.0003** | **0.001** | **0.006** | **0.03** | **0.16** | **0.80** | **4.00** | **20** | **100** |
| **SAMPLE1** | 0.728 | 0.764 | 0.714 | 0.777 | 0.753 | 0.924 | 0.981 | 0.996 | 0.611 | 0.076 |
| **SAMPLE2** | 0.906 | 0.877 | 0.901 | 0.919 | 0.838 | 0.966 | 1.065 | 1.043 | 0.632 | 0.077 |
| **SAMPLE3** | 0.944 | 0.952 | 0.960 | 0.959 | 0.944 | 1.055 | 1.243 | 1.110 | 0.656 | 0.077 |
| **M EA N** | 0.859 | 0.865 | 0.858 | 0.885 | 0.845 | 0.981 | 1.096 | 1.050 | 0.633 | 0.077 |

**Source data for Fig. S6.**

| **P24 Standard (pg/ml)** | **Kinetic OD**  **Reading** | **Kinetic OD**  **Reading** |  |  |  | |
| --- | --- | --- | --- | --- | --- | --- |
|  | **Test 1** | **Test 2** |  |  |  | |
| 1000 | 65.1 | 58.63 |  |  |  | |
| 500 | 40.61 | 37.48 |  |  |  | |
| 250 | 25.01 | 22.69 |  |  |  | |
| 125 | 14.3 | 12.94 |  |  |  | |
| 62.5 | 8.81 | 8.63 |  |  |  | |
| 31.25 | 6.27 | 5.94 |  |  |  | |
| 15.6 | 4.99 | 4.18 |  |  |  | |
| 7.8 | 3.55 | 3.59 |  |  |  | |
|  |  |  |  |  |  | |
| **Sample ID** | **Day 3 (1:10 diluton)** | | **Day 5 (1:10 diultion)** | | **Day 6 (1:100 dilution)** | |
|  | **Test 1** | **Test 2** | **Test 1** | **Test 2** | **Test 1** | **Test 2** |
| HIV Control | 6.08 | 5.88 | 55.09 | - | 24.12 | 23.51 |
| HIV Control | 8.99 | 8.44 | 58.74 | - | 27.58 | 27.25 |
| + 10 uM TIQ-15 | 8.23 | 7.93 | 49.53 | - | 21.82 | 21.15 |
| + 50 uM TIQ-15 | 8.5 | 8.44 | 16.71 | - | 7.00 | 7.01 |
