# Supplementary material for: Characterization of a CXCR4 antagonist TIQ-15 with dual tropic HIV entry inhibition properties
Source: PLoS Pathog. 2024 Aug 15;20(8):e1012448. doi: 10.1371/journal.ppat.1012448 (PMC11349218; doi:10.1371/journal.ppat.1012448)
Supplement: S1 Appendix — (DOCX) [file ppat.1012448.s009.docx]

**S1 Appendix**

These are expanded Materials and Methods:

**Isolation of resting CD4 T cells and memory CD4 T Cells from peripheral blood.** Resting CD4 T cells were purified from peripheral blood by two rounds of negative selection as previously described (1). All protocols involving human subjects were reviewed and approved by the George Mason University IRB. Briefly, for the first round of depletion, monoclonal antibodies against human CD14, CD56 and HLA-DR, DP, DQ (BD biosciences) were used. For the second round of depletion, monoclonal antibodies against human CD8, CD11b and CD19 (BD biosciences) were used. Antibody bound cells were depleted by using Dynabeads Pan Mouse IgG (Invitrogen). Purified cells were cultured in RPMI-1640 medium supplemented with 10% heat-inactivated fetal bovine serum (Invitrogen), penicillin (50U/ml) (Invitrogen), and streptomycin (50µg/ml) (Invitrogen). Cells were rested overnight before infection or treatment.

**Virus preparation and infection of cells.** Primary and transformed T cells are cultured in RPMI-1640 medium supplemented with 10% heat-inactivated fetal bovine serum (Invitrogen), penicillin (50U/ml) (Invitrogen), and streptomycin (50µg/ml) (Invitrogen). Virus stocks of HIV-1(NL4-3), BlaM-Vpr containing HIV-1(NL4-3), and VSV-G-pseudotyped HIV were prepared as described previously (1, 2). Briefly, HEK293T cells were transfected using Lipofectamine 2000 (Invitrogen) or Transfectin (Virongy) with cloned proviral DNA. Supernatant was harvested at 48 hours, and filtered through a 0.45-µm nitrocellulose membrane. Levels of p24 in the viral supernatant were measured in with ELISA using an in-house ELISA kit. Virus titer (TCID50) was measured by infection of a Rev-dependent GFP indicator cell line, Rev-A3R5-GFP (3-6). For HIV infection of Rev-CEM-GFP-Luc cells, cells (2x10^5^/0.1 ml) were incubated with HIV for 2 hours at 37^o^C, and then washed twice with medium to remove unbound virus. Infected cells were resuspended in fresh medium (2x10^5^/ml) and cultured for 48 to 72 hours. HIV infection was measured by Flow-cytometry (FACS Calibur, BD Biosciences) for GFP positive cells. To exclude drug cytotoxicity, propidum iodide (PI) (2 µg/ml, Fluka) was added into the cell suspension prior to flow cytometry, and only viable cell population (PI negative) was used for quantifying GFP expression. Alternatively, cells were also collected and resuspended in 0.1 ml of luciferase assay buffer (Promega), and luciferase activity was measured in live cells using a GloMax-Multi Detection System (Promega). For infection of blood resting CD4 T cells, cells (1x10^6/^ml) were infected with HIV for 2 hours at 37^o^C, and then washed twice with medium to remove unbound virus. Infected cells were resuspended into fresh medium (1x10^6^/ml) and cultured for 5 days without stimulation. In occasion, IL-7 (1 ng/ml) was added every two days untill day 5. Cells were activated at day 3 or 5 with anti-CD3/CD28 magnetic beads at 4 beads per cell as described previously (1). For quantifying viral replication, culture supernatant (100 µl) was taken every two days after stimulation and used for p24 ELISA using an in-house ELISA kit. Plates were kinetically read using an ELx808 automatic microplate reader (Bio-Tek Instruments) at 630nm. For pretreatment of cells with compounds, unless specified, cells were pretreated with TIQ-15, AMD3100 or Dimethyl sulfoxide (DMSO) (1%, as control) for 1 hour at 37oC, and then infected with HIV. TIQ-15 was synthesized as previously described by Truax et. al. (7). AMD3100 and maraviroc were purchased from commercial vendors.

**Infection of PBMCs by HIV clinical isolates and TIQ-15 inhibitio**n. Fresh human PBMC (Biological Specialty Corporation, Colmar, PA) (1-2 x 10^6^ cells/ml) were cultured in RPMI 1640 + 15 % FBS medium with 4 µg/mL Phytohemagglutinin (PHA) (Sigma, St. Louis, MO) and 20 U/mL recombinant human IL-2 (R&D Systems Inc., Minneapolis, MN) for 48-72 hours at 37^o^C. Cells were plated in 96 well round bottom microplate at 50 μL/well (5 x 104 cells/well). TIQ-15 dilutions were prepared in microtiter tubes, and 100 μL of each concentration was added to each test well, and 50 μL of a predetermined dilution of virus (final MOI, 0.1) was added. The PBMC cultures were maintained for seven days following infection at 37°C, 5% CO2. After this period, cell-free supernatant samples were collected for analysis of reverse transcriptase activity, and compound cytotoxicity was measured by additional MTS assay.

**Synergy studies of TIQ-15 with maraviroc.** Total cell and viability quantification were performed using a hemacytometer and trypan blue exclusion. Cell viability is greater than 95% for the cells to be utilized in the assay. HIV‐1(IIIB) and HIV-1(Ba‐L) were obtained from the NIH AIDS Research and Reference Reagent Program. For each assay, a pre-titered aliquot of virus (0.001 TCID50/cell) was used in a 96 well plate. A checkerboard plate format was used to test five concentrations of drug A (maraviroc) in all possible combinations with eight concentrations of drug B (TIQ‐15). Combination antiviral efficacy was evaluated on three identical assay plates (i.e., triplicate measurements) that include cell control wells (cells only) and virus control wells (cells plus virus). Combination cytotoxicity was evaluated in parallel on two identical assay plates (i.e., duplicate measurements) that include cell control wells. A compound color control plate was included from background subtraction when color is observed at the concentrations of compound used in the experiments. Antiviral efficacy was measured as the inhibition of β‐galactosidase expression and cytotoxicity was monitored by MTS staining at the experimental endpoint. For data analysis, the data was analyzed according to the method of Prichard and Shipman (8), using the MacSynergy II program for data analysis and statistical evaluation. Briefly, the MacSynergy II program calculates the theoretical additive interactions of the drugs based on the Bliss Independence mathematical definition of expected effects for drug‐drug interactions. The Bliss Independence model is based on statistical probability and assumes that the drugs act independently to affect virus replication; this Independent Effects model is also referred to as a Dual‐Site (DS) model and was used for all combination analyses reported herein. Theoretical additive interactions were calculated from the dose response curves for each drug used individually. This calculated additive surface, which represents predicted or additive interactions, was then subtracted from the experimentally determined dose‐response surface to reveal regions of non‐additive activity. The resulting surface would appear as a horizontal plane at 0% inhibition above calculated if the interactions were merely additive. Any peaks above this plane-of-additivity would be indicative of synergy. Similarly, any depressions below the plane-of-additivity would indicate antagonism. The 95% confidence intervals around the experimental dose‐response surface were used to evaluate the data statistically and the volume of the peaks/depressions is calculated and used to quantify the volume of synergy/antagonism produced. The volume of the peaks observed in the synergy plots (in units of concentration times and concentration times percent; *e.g.* μM^2^%, nM^2^%, nMμM%, etc.) was calculated by the program. This peak volume is the three‐dimensional counterpart of the area under a 3‐dimensional dose response surface and is a quantitative measure of synergy or antagonism. For these studies, synergy is defined as drug combinations yielding synergy volumes greater than 50. Slightly synergistic activity and highly synergistic activity have been operationally defined as yielding synergy volumes of 50‐100 and >100, respectively. Additive drug interactions have synergy volumes in the range of ‐50 to 50, while synergy volumes between ‐50 and ‐100 are considered slightly antagonistic and those < -100 are highly antagonistic.

**Conjugation of antibodies to magnetic beads and stimulation of resting CD4 T cells.** Monoclonal antibodies against human CD3 (clone UCHT1), CD28 (clone CD28.2) were purchased from BD Biosciences. For conjugation, 10 µg of antibodies were conjugated to 4x10^8^ Dynabeads Pan Mouse IgG (Invitrogen) for 30 minutes at room temperature. Magnetic beads were washed with PBS and resuspended in 1 ml of PBS 0.1%BSA and used to stimulate resting CD4 T cells as previously described (1).

**Surface staining of CD4, CXCR4 and CCR5.** Resting CD4 or A3R5 CD4 T cells were stained with FITC-labeled monoclonal antibody against human CD4 (clone PRA-T4) or PE/Cy5-labeled monoclonal antibody against human CXCR4 (clone 12G5) (BD Biosciences) or PE-Cy5-labeled monoclonal antibody against CCR5 (Clone-J418F1, Biolegend). Cells were stained on ice in PBS + 0.1% BSA for 30 minutes, washed with cold PBS + 0.1% BSA, and then analyzed on a FACSCalibur (BD Biosciences).

**Viral Entry Assays.** The BlaM-Vpr-based viral entry assay was performed as previously described (1, 9). Briefly, viruses were generated by co-transfection of HEK293T cells with three plasmids: pNL4-3, pAdvantage (Promega) and pCMV4-3BlaM-Vpr (kindly provided by Dr. Warner C. Greene) (at a ratio of 6:1:2). Supernatant was harvested at 48 hours posttransfection, concentrated, and then used for infection. CEM-SS cells (1x10^6^) were infected with BlaM-Vpr containing viruses at 37oC for 4 hours. Cells were washed and then loaded with CCF2 for flow cytometry, which was performed using a Becton Dickinson LSR II (Becton Dickinson). β-lactamase and CCF2 measurements were performed using a 407-nm violet laser with emission filters of 525/50 nm (green fluorescence) and 440/40 nm (blue fluorescence), respectively. Green and blue emission spectra were separated using a 505LP dichroic mirror. The UV laser was turned off during the analysis. Flow cytometry was also performed using Cytek Northern Lights (Cytek Biosciences) Data analysis was performed using FlowJo software (FlowJo).

**Real time PCR amplification of viral DNA.** Viral total DNA was measured by real time PCR as described previously (50). Briefly, infected resting CD4 T cells were directly lysed in DNA extraction lysis buffer (SV Genomic DNA Isolation System, Promega). Total cellular DNA was extracted and quantitative real time PCR analyses of viral total DNA were carried out using the Bio-Rad iQ5 real time PCR detection system, utilizing the forward primer 5’-LTR-U5, the reverse primer 3’-gag, and the probe FAM-U5/gag (1, 10, 11). Pre-quantified, full-length proviral plasmid pHIV(NL4-3) was used as the DNA standard.

Chemotaxis assay. One half million resting CD4 T cells were pretreated with TIQ-15 or DMSO (1%, as control), resuspended into 100 µl of RPMI-1640 medium, and then added to the upper chamber of a transwell plate (6.5 mm diameter and 5 µm pore size with a polycarbonate membrane) (Corning). The lower chamber was filled with 600 µl of medium premixed with 50 nM SDF-1 (R&D systems). The transwell plate was incubated at 37oC for 1 hour, and then the upper chamber was removed and cells in the lower chamber were counted in a Beckman coulter Z2 cell and particle counter.

**Intracellular staining of p-cofilin.** One half million cells pretreated with TIQ-15 (10 µM) or DMSO (1%, as control) for 1 hour at 37oC. Cells were stimulated with SDF-1 for various periods of time. Cells were fixed, permeabilized, washed, and then stained with Rabbit anti-human p-cofilin antibody (1:50 dilution) for 60 min at room temperature using Intracellular Protein Staining kit (Virongy). Cells were washed twice and stained with Alexa Fluor 488-labeled chicken anti-rabbit antibodies (1:2500 dilution) (Invitrogen, Carlsbad, CA) for 30 minutes in dark at room temperature. Cells were washed twice, and then analyzed on FACSCalibur (BD Biosciences).

**cAMP Assay.** cAMP production was assayed as previously described (32). CHO-Glo cells, a cell line stably expressing the GloSensor cAMP biosensor (Promega) were transfected with cDNA encoding human CXCR4 in pIRES-AcGFP1 (Clontech), and a stable cell line was isolated (CXCR4-Glo cells). Cells were grown to confluency on 96-well, white-walled, clear-bottom plates (Corning). cAMP production was initiated with 1 μM forskolin (Tocris) in Locke’s buffer containing D-luciferin (Gold Biotechnology) and vehicle or a fixed 28 nM (EC80 value) concentration of SDF-1α (R&D Systems) and the indicated compound. Sixteen minutes after drug treatment, luminescence was measured using the EnVision Multilabel Plate Reader (Perkin-Elmer). EC50 and IC50 values were calculated from the 4-parameter logistic equation.

**HIV-NefM1 Induced Mitochondrial Membrane Depolarization Assay.** The 11-mer peptide Nef Motif-1 (NefM1, TNAACAWLEAQ) was obtained from Sigma Genosys. For the depolarization assay, 5,5’,6,6’-tetrachloro-1,1’,3,3’-tetraethyl-benzimidazolylcarbocyanine iodide (JC-1; CBIC) was obtained from Invitrogen. Jurkat cells were treated with 10 ng/ml of NefM1 and different doses of AMD3100 (50, 200, 400, 600, 800, and 1000 nM), maraviroc (0.1, 1, 10, 100, 1000, and 10,000) or AZT (0.1, 1, 10, 100, and 1000 nM) for 24 hours. The cultures were then washed in 1 x PBS and 100 µl of fresh JC-1 stain solution (a stock solution of JC-1 at a concentration of 2.5 mg/ml in DMSO was diluted into culture media to a final concentration of 10 µg/ml) was added to the cell pellet and incubated at 37 ᵒC for 10 min. The cells were washed in 1 x PBS, and rinsed in imaging buffer (High K+ buffer, 137 mM KCl, 3.6 mM NaCl, 0.5 mM MgCl2, 1.8 mM CaCl2, 1.6 mM NaH2PO4 and 4.3 mM NaHCO3, pH 7.4). Stained cultures were viewed immediately by epifluorescence on a computer-controlled microscope system (Carl Zeiss, Thornwood, NY) and images were captured via a charge-coupled device camera (MC 100 SPOT 60910; Photonic Science, East Sussex, United Kingdom). Further image processing was conducted with Image-Pro 2.0 software (Media Cybernetics). SigmaPlot 10 was used for numerical and graphical analyses of all data obtained.

**β‐galactosidase chemiluminescent endpoint analysis.** A chemiluminescent endpoint was used to determine the extent of β‐galactosidase expression as a measure of HIV‐1 infection. At 2 days post infection, plates were aspirated and PBS was added to each well. Gal‐screen™ reagent (Life Technologies) was then added per the manufacturer’s instructions for chemiluminescent detection of β‐galactosidase activity and incubated at room temperature for 90 minutes. The resulting chemiluminescence signal was then read using a Microbeta Trilux luminescence reader (PerkinElmer/Wallac).

**MTS staining for cell viability.** At assay termination, plates were stained with the soluble tetrazolium‐based dye MTS (CellTiter 96 Reagent, Promega) to determine cell viability and quantify compound toxicity. 15 μL of MTS reagent was added per well and the microtiter plates were incubated for 1.5‐2 hours at 37°C, 5% CO2 to assess cell viability. Adhesive plate sealers were used in place of the lids and the plates are read spectrophotometrically at 490/650 nm with a SpectraMax i3 plate reader (Molecular Devices).

**References**

1. Yoder A, Yu D, Dong L, Iyer SR, Xu X, Kelly J, et al. HIV envelope-CXCR4 signaling activates cofilin to overcome cortical actin restriction in resting CD4 T cells. Cell. 2008;134(5):782-92.

2. Yu D, Wang W, Yoder A, Spear M, Wu Y. The HIV envelope but not VSV glycoprotein is capable of mediating HIV latent infection of resting CD4 T cells. PLoS Pathog. 2009;5(10):e1000633.

3. Wu Y, Beddall MH, Marsh JW. Rev-dependent indicator T cell line. Current HIV Research. 2007;5:395-403.

4. Wu Y, Beddall MH, Marsh JW. Rev-dependent lentiviral expression vector. Retrovirology. 2007;4(1):12.

5. Fu Y, He S, Waheed AA, Dabbagh D, Zhou Z, Trinite B, et al. PSGL-1 restricts HIV-1 infectivity by blocking virus particle attachment to target cells. Proc Natl Acad Sci U S A. 2020;117(17):9537-45.

6. Liu Y, Fu Y, Wang Q, Li M, Zhou Z, Dabbagh D, et al. Proteomic profiling of HIV-1 infection of human CD4(+) T cells identifies PSGL-1 as an HIV restriction factor. Nat Microbiol. 2019;4(5):813-25.

7. Truax VM, Zhao H, Katzman BM, Prosser AR, Alcaraz AA, Saindane MT, et al. Discovery of tetrahydroisoquinoline-based CXCR4 antagonists. ACS medicinal chemistry letters [Internet]. 2013 2013/11//; 4(11):[1025-30 pp.].

8. Prichard MN, Shipman C, Jr. A three-dimensional model to analyze drug-drug interactions. Antiviral Res. 1990;14(4-5):181-205.

9. Cavrois M, De Noronha C, Greene WC. A sensitive and specific enzyme-based assay detecting HIV-1 virion fusion in primary T lymphocytes. Nat Biotechnol. 2002;20(11):1151-4.

10. Kelly J, Beddall MH, Yu D, Iyer SR, Marsh JW, Wu Y. Human macrophages support persistent transcription from unintegrated HIV-1 DNA. Virology. 2008;372:300-12.

11. Wu Y, Marsh JW. Early transcription from nonintegrated DNA in human immunodeficiency virus infection. J Virol. 2003;77(19):10376-82.
