# Supplementary material for: Characterization of a CXCR4 antagonist TIQ-15 with dual tropic HIV entry inhibition properties
Source: PLoS Pathog. 2024 Aug 15;20(8):e1012448. doi: 10.1371/journal.ppat.1012448 (PMC11349218; doi:10.1371/journal.ppat.1012448)
Supplement: Attachment — Submitted filename: ZhouResponses to reviewers 061824.pdf [file ppat.1012448.s010.pdf]

Ref: **PPATHOGENS-D-24-00469**
Characterization of a CXCR4 antagonist TIQ-15 with dual tropic HIV entry inhibition properties

June 18, 2024

Editor
PLoS Pathogens

Dear Editor:

We hereby resubmit our revised manuscript entitled: “Characterization of a CXCR4 antagonist TIQ-15 with dual tropic HIV entry inhibition properties” by Zhou et al., to be considered for publication in *PLoS Pathogens*.

In this revision, we have performed additional experiments (new **Fig 4A, 4G**, and **Fig. S5 to S6**) to address the reviewers’ suggestions and comments. A detailed point-by-point response to reviewers’ comments is attached below.

In this revision, we also added three new co-authors: Sameer Tiwari, who conducted the new TIQ-15-mediated CXCR4/CCR5 downregulation assays, and the high-dosage HIV(AD8) infection experiments; Amrita Haikerwal, who performed the R5 viral entry assay; Yang Han, who performed R5 HIV latent infection of blood resting CD4 T cells.

We would like to thank the editor for giving the opportunity to revise our manuscript, and the reviewers for their insightful and constructive suggestions. We believe that the manuscript has been significantly improved with these new studies.

We thank you for your consideration!

Yuntao Wu, Ph.D, Professor
Center for Infectious Disease Research/George Mason University
Tel: (703) 993 4299; Email: ywu8@gmu.edu

Lawrence J. Wilson
Department of Chemistry/Emory University
Email: ljwilso@emory.edu

Detailed Responses to Reviewer's Comments:

Part I - Summary

Reviewer #1*: The investigators here previously identified a chemical inhibitor of CXCR4-tropic HIV-1 that is further characterized in this report. Most of the data here establish the inhibition of CXCRF by TQ-15, including blocking SDF-1 binding, downmodulation of surface CXCR4, and prevention of entry of X4 HIV-1 isolates into cells. These data seem reasonable. However, there is also an effect, albeit weaker, on the replication of R5 virus, and this is then put forth as an important characteristic of the chemical. There is little characterization of how TQ-15 inhibits R5 tropic HIV. Without a mechanism or step in the viral lifecycle identified, this report doesn’t rule out that this is merely an*

off-target effect or toxicity of the chemical that is not picked up in the cytotoxicity assay. Novelty here relies on the R5 virus mechanism of inhibition.

Responses: We appreciate the insightful comments of the reviewer, and conducted additional experiments as suggested. New data are now presented in **new Fig. 4G**, **Fig. S4 to S6.** First, we repeated the experiment in the inhibition of viral infection using higher dosages of HIV(AD8) virus (infecting 4% and 14% cells). We also used propidium iodide (PI) staining of dead cells to monitor cytotoxicity. HIV replication (GFP expression) was quantified only in the viable cell population. We found that at the higher viral inocula, at 10 μM, TIQ-15 minimally inhibited HIV-1(AD8), and at 50 μM, TIQ-15 only reduced HIV(AD8) replication to around 50% (**Fig. S4**), which is in great contrast to TIQ-15's potent inhibition of the X4 viruses at 13.3 nM (IC50). We also followed viral infection steps. Our new data revealed that TIQ-15 does not downregulate CCR5 even at 50 μM (**new Fig. 4G**), which is in great contrast of the potent downregulation of CXCR4 by TIQ-15 (new **Fig. 4A**). In addition, we performed viral entry assay and found that TIQ-15 only moderately inhibited HIV(AD8) entry at 10-50 μM (**Fig. S5**), which is also in great contract to the complete inhibition of the T-tropic HIV(NL4-3) entry by TIQ-15 and AMD3100 (**Fig. 4D**). These new results demonstrate that TIQ-15 inhibition of R5 virus at high dosages was not a result of cytotoxicity, it moderately inhibited viral entry.

Reviewer #2: *Manuscript by Zhou et al describes studies characterizing small molecule TIQ-15 on suppression of X4-tropic HIV-1 infection. In a previous publication, TIQ-15 was identified as a CXCR4 antagonist and shown to inhibit X4-tropic HIV-1 infection, compete with SDF-1 in a binding assay, and prevent cAMP production. In this manuscript, the actions of TIQ-15 were further studied. Authors found that TIQ-15 inhibits SDF-1a/CXCR4 mediated chemotaxis and T cell migration and interferes with apoptosis induced by NefM1 peptide-CXCR4 interaction. The inhibition of HIV-1 infection was further probed, and they found that 1) CXCR4 is down-regulated upon treating cells with 10 μM TIQ-15, 2) TIQ-15 can inhibit R5- or dual-tropic virus infection albeit at higher IC50 values, and 3) TIQ-15 acts synergistically with Maraviroc to block a R5-tropic virus replication.*
The strengths of the manuscript are that TIQ-15 is a lead that targets HIV-1 entry using the CXCR4 coreceptor; currently there is no approved antiviral against this target. Additionally, the synergistic effect with Maraviroc is interesting and intriguing. However, the mechanism(s) by which TIQ-15 inhibits X4- or R5-mediated entry is not addressed. The down regulation of CXCR4 could potentially explain inhibition of X4-tropic virus entry. However, it is unclear how this small molecule inhibits R5-tropic virus infection.

Responses: we appreciate all the positive comments and the insightful suggestions. We conducted additional experiments in the inhibition of R5-tropic viral entry and downregulation of CCR5 by TIQ-15, as suggested. New data are now presented in **new Fig. 4G**, **Fig. S4 to S6.** We found that TIQ-15 does not downregulate CCR5 even at 50 μM (**new Fig. 4G**), which is in great contrast of the potent downregulation of CXCR4 by TIQ-15 at 10 μM (**Fig. 4A**). In addition, we performed viral entry assay and found that TIQ-15 only moderately inhibited HIV(AD8) entry at 10-50 μM (**Fig. S5**), which is also in great contract to the complete inhibition of the T-tropic HIV(NL4-3) entry by TIQ-15 and AMD3100 (**Fig. 4D**).

We also performed a TIQ-15 dosage-dependent downregulation of CXCR4 on CD4 T cells (**new Fig. 4A)** and found that TIQ-15 downregulates CXCR4 in a dosage-dependent manner, which downregulates CXCR4 around 50% at 10 uM, but minimally downregulates CXCR4 at dosages below 10 nM. Given that TIQ-15 inhibits X4-tropic HIV with a IC50 of 13.3 nM (**Fig. 3B**), these results do not support the hypothesis that downregulation of CXCR4 is the primary antiviral mechanism of TIQ-15.

The pharmacology of TIQ-15 as a CXCR4 antagonist has also been tested previously. The data has shown that this molecule inhibits the function of the receptor in many different assays including calcium flux (IC50 = 5-10 nM), ^{125}I-SDF binding (IC50 = 112 nM) and beta-arrestin recruitment inhibition (IC_{50} = 19 nM). The data in this paper (cAMP, IC50 = 41 nM; chemotaxis, IC50 = 176 nM) provides similar outcomes showing that TIQ-15 binds to and blocks the natural ligand SDF-1 from acting on the receptor in a similar concentration range. **Furthermore, TIQ-15 treatment in a calcium flux assay absent of SDF-1 shows no effects indicating that TIQ-15 is not a CXCR4 agonist.** The data showing blockade of SDF-1 receptor internalization via the beta-arrestin pathway is similar in potency to the other assays of blocking receptor signaling (Calcium flux, cAMP; 19 versus 10 & 41 nM) indicating that **TIQ-15 blocks receptor internalization via affecting a signaling pathway.**

The experiment showing reduction of CXCR4 receptor population by TIQ-15 at a 10,000 nM concentration indicates a different property. Here about 50% of the receptor is absent from the surface and there was no SDF-1 used in the experiment. This result can be interpreted to be a TIQ-15 promoted receptor internalization/downregulation but this occurs at a much higher concentration than inhibition of signaling (10-50 nM versus 10,000 nM). It could be that TIQ-15 affects CXCR4 receptor surface dimerization or receptor recycling at higher concentrations apart from and independent of blockade of receptor signaling. However, this experiment is more likely a unique and under reported effect rather than a rebuke to the characterization of TIQ-15 as a CXCR4 receptor antagonist. For example, AMD3100 has been reported to cause CXCR4 receptor tolerance in certain cell types (Hitchinson et al. Biased antagonism of CXCR4 avoids antagonist tolerance. ***Sci Signal.*** 2018 Oct 16;11(552):eaat2214. doi: 10.1126/scisignal.aat2214). This could be a similar effect with TIQ-15 in this assay.

Part II: Major Issues - Key Experiments Required for Acceptance

Reviewer #1*: Therefore to make claims that this will be a useful dual-tropic inhibitor, more data on inhibition of R5 virus is needed.*

Responses: We have conducted additional experiments on R5 virus. New data are now presented in **new Fig. 4G**, **Fig. S4 to S6.**

Specific criticism:

1) Figure 2 shows an effect of TIQ-15 on T cell polarization. It would be helpful to provide representative images of polarized vs. depolarized cells as assessed by this assay. It was hard to tell from the description what was being assessed, but presumably this is mitochondrial membrane depolarization where depolarization is indicated by a decrease in the red/green fluorescence intensity profile. Please clarify and include in methods.

Responses: we have included the fluorescent images of the assay in new revised **Fig.2.**

2) Figure 3 uses X4 tropic virus to examine inhibition of infection of CD4+ T cells. Given the focus on dual-tropic inhibition, this figure would benefit from the use of an R5 tropic strain using the same assay.

Responses: We repeated the experiments on the inhibition of R5 HIV replication by TIQ-15. The new results are presented in **new Fig. 4G**, **Fig. S4 to S6.** These new results demonstrate that TIQ-15 inhibition of R5 virus at high dosages (50 uM).

3) Figure 4F provides some evidence of inhibition of R5 virus. The control infection rate was quite low, and the effect shown for higher MOI would be useful in judging the actual decrease in infection by R5 virus.

Responses: We repeated Fig. 4F using higher dosages of HIV(AD8) (4% and 14%). New results are now shown in **Fig. S4**.

4) Figure 5 is the best example of data showing inhibition of dual tropic and R5 virus along with X4 virus. However, a one-time measurement of RT from supernatants could be complemented by studies to understand where the block in replication is taking place. For R5 virus, is it entry, post-entry, transcription, translation, assembly? The step is not established here, and if it was it would help in understanding if this is a block that is really specific for R5 virus or a general effect on cells that reduces cell health enough to reduce particle production.

Responses: We appreciate the insightful comments of the reviewer, and conducted additional experiments as suggested. New data are now presented in **new Fig. 4G**, **Fig. S4 to S6.** First, we used propidium iodide (PI) staining to monitor cytotoxicity. HIV replication (GFP expression) was quantified only in the viable cell population. We found that at the higher viral inocula, at 10 μM, TIQ-15 minimally inhibited HIV-1(AD8), and at 50 μM, TIQ-15 only reduced HIV(AD8) replication to around 50% (**Fig. S4**), which is in great contrast to TIQ-15's potent inhibition of the X4 viruses at 13.3 nM (IC50). We also followed viral infection steps. Our new data revealed that TIQ-15 does not downregulate CCR5 even at 50 μM (**new Fig. 4G**), which is in great contrast of the potent downregulation of CXCR4 by TIQ-15 at 10 μM (**Fig. 4A**). In addition, we performed viral entry assay and found that TIQ-15 moderately inhibited HIV(AD8) entry at 10-50 μM (**Fig. S5**), which is also in great contract to the complete inhibition of the T-tropic HIV(NL4-3) entry by TIQ-15 and AMD3100 (**Fig. 4D**). These new results suggest that TIQ-15 inhibition of R5 virus at high dosages was not a result of cytotoxicity, it moderately inhibited viral entry.

Reviewer #2: *1. How does TIQ-15 block X4 virus infection? In a previous study, it was hypothesized that the small molecule binds a pocket in CXCR4. Unfortunately, the current report does not add more insights to the mechanism of action of TIQ-15. A single data point in **Fig. 4A** shows that at 10 μM, TIQ-15 reduces CXCR4 expression on T cell surface.*

However, it is unclear that CXCR4 down regulation is responsible for blocking HIV-1 infection at the IC50 or IC90 concentrations. Examining the effect of TIQ-15 treatment on cell surface CXCR4 expression using multiple concentrations and time points may help address whether X4 down regulation is a mechanism of action in inhibiting X4-tropic virus infection.

Response: We performed a TIQ-15 dosage-dependent downregulation of CXCR4 on CD4 T cells (**new Fig. 4A)** and found that TIQ-15 downregulates CXCR4 in a dosage-dependent manner, which downregulates CXCR4 around 50% at 10 uM, but minimally downregulates CXCR4 at dosages below 10 nM. Given that TIQ-15 inhibits X4-tropic HIV with a IC50 of 13.3 nM (**Fig. 3B**), these results do not support the hypothesis that downregulation of CXCR4 is the primary antiviral mechanism of TIQ-15.

The pharmacology of TIQ-15 as a CXCR4 antagonist has been tested previously. The data has shown that this molecule inhibits the function of the receptor in many different assays including calcium flux (IC50 = 5-10 nM), ^{125}I-SDF binding (IC50 = 112 nM) and beta-arrestin recruitment inhibition (IC_{50} = 19 nM). The data in this paper (cAMP, IC50 = 41 nM; chemotaxis, IC50 = 176 nM) provides similar outcomes showing that TIQ-15 binds to and blocks the natural ligand SDF-1 from acting on the receptor in a similar concentration range. **Furthermore, TIQ-15 treatment in a calcium flux assay absent of SDF-1 shows no effects indicating that TIQ-15 is not a CXCR4 agonist.** The data showing blockade of SDF-1 receptor internalization via the beta-arrestin pathway is similar in potency to the other assays of blocking receptor signaling (Calcium flux, cAMP; 19 versus 10 & 41 nM) indicating that **TIQ-15 blocks receptor internalization via affecting a signaling pathway.**

The experiment showing downregulation of CXCR4 receptor population by TIQ-15 at a 10,000 nM concentration indicates a different property. Here about 50% of the receptor is absent from the surface and there was no SDF-1 used in the experiment. This result can be interpreted to be a TIQ-15 promoted receptor internalization/downregulation but this occurs at a much higher concentration than inhibition of signaling (10-50 nM versus 10,000 nM). It could be that TIQ-15 affects CXCR4 receptor surface dimerization or receptor recycling at higher concentrations apart from and independent of blockade of receptor signaling. However, this experiment is more likely a unique and under reported effect rather than a rebuke to the characterization of TIQ-15 as a CXCR4 receptor antagonist. For example, AMD3100 has been reported to cause CXCR4 receptor tolerance in certain cell types (Hitchinson et al. Biased antagonism of CXCR4 avoids antagonist tolerance. ***Sci Signal.*** 2018 Oct 16;11(552):eaat2214. doi: 10.1126/scisignal.aat2214). This could be a similar effect with TIQ-15 in this assay.

2. Given its effect on R5-tropic virus, perhaps examining CCR5 expression upon treatment of TIQ-15 will be fruitful.

Response: We have performed additional experiments in TIQ-15 on CCR5 expression. New results are now presented in **new Fig. 4G**.

Part III – Minor Issues: Editorial and Data Presentation Modifications

Reviewer #2: *1. Results from Fig. 5 and Table 1 are inconsistent. Especially for 00KE_KER2008, Figure 5 listed the IC50 as 71.38 nM whereas Table 1 listed the IC50 as 660 nM.*

Response: We corrected the typo in Table 1, and recalculated and checked all the data in New **Table 1**.
